# Environmentally Safe Synthesis of Gold Nanoparticles for Antigen Detection in CLIA System

**DOI:** 10.1021/acsomega.5c07361

**Published:** 2025-10-08

**Authors:** Keilla Gomes Machado, Luiza Felippi de Lima, Wellington Vieira de Souza, Raquel Checca, Daniel R. Louzada, Rubem L. Sommer, André Felipe Streck, Cesar Aguzzoli, Mariana Roesch-Ely

**Affiliations:** † 58802 Instituto de Biotecnologia—Universidade de Caxias do Sul, Caxias do Sul 95070-560, Brazil; ‡ PPGMAT- Universidade de Caxias do Sul, Caxias do Sul 95.070-560, Brazil; § 74350 Centro Brasileiro de Pesquisas Físicas, Rio de Janeiro 22.290-180, Brazil

## Abstract

The use of gold nanoparticles (AuNPs) has been explored in the last decades, and most recently, efforts have been made to associate their production with environmentally safe routes and biotechnological processes. The versatility of bioconjugation strategies used for AuNPs makes them a promising tool for developing alternative detection methods for traditional diagnostic assays. In this work, we present a chemiluminescent immunoassay based on AuNPs for specific and sensitive detection of SARS-CoV-2. The AuNPs were processed through a physical route, deposited on a solid substrate via magnetron sputtering, and functionalized with anti-SARS-CoV-2 antibodies. The colloidal stability of the AuNPs was monitored through UV–vis spectroscopy and transmission electron microscopy (TEM). UV–vis showed a plasmon resonance peak at ∼535 nm, consistent with the TEM results, which presented an average size of the AuNP around 40 nm. Altogether, nasopharyngeal swabs of 180 patients were tested with the antibody-AuNP bioconjugates to recognize their corresponding antigens through chemiluminescent immunoassays (CLIA). To assess the affinity and specificity of the functionalized AuNPs, comparative experiments of CLIA and classical enzyme-linked immunosorbent assay (ELISA) were conducted. CLIA results outperformed ELISA and were effective in detecting SARS-CoV-2–positive samples with a viral load exceeding 1.19 ng/mL. Analysis of the Receiver Operating Characteristic curve yielded an AUROC of 0.909 (95% CI: 0.864–0.955), indicating excellent discriminative ability, with a sensitivity of 91% and a specificity of 85%. The results provided new insights into AuNPs produced by an environmentally safe synthesis with excellent functionalization and detection capability for diagnostic applications.

## Graphical abstract

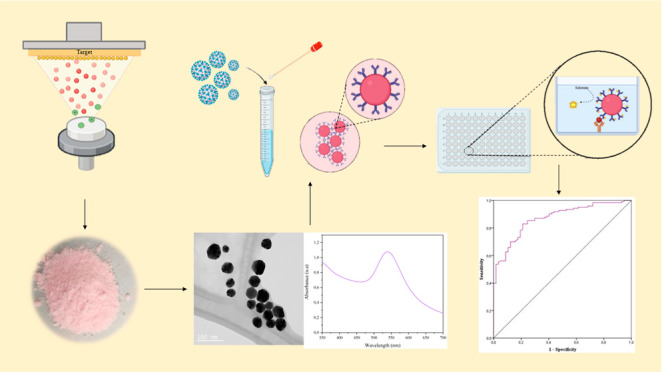

## Introduction

Studies related to the synthesis and application of gold nanoparticles (AuNPs) have shown exponential growth in recent years, particularly due to the search for more sustainable alternatives that reduce waste and environmental impact.^1^ Bottom-up approaches, such as the use of plant extracts as reducing and stabilizing agents, have been explored,^2^ as well as top-down techniques, such as magnetron sputtering deposition. In this latter method, Au is deposited on liquid polyethylene glycol (PEG) or other substrates, generating clean NPs since no chemical reactions, stabilizing agents, surfactants, or chemical residues are involved, thereby avoiding contamination.^1,3–6^

AuNPs exhibit unique properties, including the possibility of synthesizing different morphologies (nanospheres, nanocubes, nanorods, nanostars, and nanocages) and functionalizing them with a wide range of biomolecules (probes, polymers, and antibodies). These characteristics have driven their use in biotechnology and healthcare, especially for therapeutic and diagnostic applications.^7–11^ One of the main advantages of using AuNP lies in their potential as signal amplifiers in assays, since the immobilization of biomolecules on their surface increases the sensitivity in the detection of disease markers. Complementary strategies, such as the use of tyramide signal amplification or horseradish peroxidase (HRP), are also employed to enhance biomarker detection at low levels.^12^ In this study, AuNPs functionalized with HRP-conjugated antibodies were used for signal amplification.

The detection of protein biomarkers remains challenging, as they are often present at very low concentrations and mixed with other macromolecules in biological fluids.^13^ To ensure specificity, assays employ affinity ligands, such as antibodies and aptamers, which recognize target proteins and mediate signal generation. In many cases, the combination of different markers is required to improve diagnostic accuracy.^14,15^ These assays, generally referred to as immunoassays,^16^ include the enzyme-linked immunosorbent assay (ELISA), widely used in laboratories for biomarker detection. However, its limited sensitivity in detecting proteins at very low concentrations may restrict clinical applications, encouraging the development of alternative methods such as chemiluminescent immunoassays (CLIA), which provide higher sensitivity.^17,18^ The incorporation of NPs into these assays has been well documented, yielding significant improvements in both sensitivity and specificity.^13,19,20^

ELISA emerges as a preferred clinical laboratory test due to its ability to accurately quantify analyte concentration; however, this technique has limitations, such as protocols with long detection time and high labor costs, which can hinder its practical application. Consequently, new techniques are being explored to replace this test,^17^ such as CLIA, which combine principles of chemiluminescence and immunoassay.^21^ CLIA offers several advantages, including a wider dynamic range, high signal intensity, absence of interfering emissions, rapid analytical signal acquisition, high stability of the reagents and their conjugates, low reagent consumption, and complete compatibility with immunological assay protocols.

In this context, the diagnosis of viral infections in humans is of particular interest, given their high prevalence and impact on public health. The recent outbreak of the SARS-CoV-2 virus, which was responsible for the COVID-19 pandemic, highlighted the urgent need for rapid and sensitive diagnostic methods. This disease has resulted in more than 759 million confirmed cases and approximately six million deaths worldwide. Although the World Health Organization declared the end of the SARS-CoV-2 pandemic in May 2023,^22–25^ viral variants continue to circulate, requiring ongoing epidemiological monitoring.^26–28^

The main methodologies for detecting SARS-CoV-2 involve identifying viral nucleic acids (molecular tests), viral proteins (antigen tests), and anti-SARS-CoV-2 antibodies (serological tests).^29,30^ Among these, the direct detection of viral proteins is particularly relevant, as it provides information about the infectious status of individuals, supporting clinical decision-making and the control of viral spread. Over the past two decades, significant advances have been achieved in the development of diagnostic methods based on AuNPs conjugated with antibodies, applicable not only to SARS-CoV-2 but also to other pathogens.^31,32^ There are several studies investigating the functionalization of antibodies using AuNPs and bringing alternatives for biomolecule loading, most of them focusing on adsorption and covalent techniques. AuNP probes prepared by direct adsorption were the most effective method presented by.^33^ Few articles, however, are exploring functionalization using a pool of antibodies as presented here, which brings advantages, especially when mixing monoclonal and polyclonal antibodies. Monoclonal antibodies offer high reproducibility and defined specificity, while polyclonal provide broader reactivity and enhanced signal due to multiepitope recognition.^34^

Accordingly, this study presents a chemiluminescent immunoassay based on AuNPs for the sensitive and specific detection of SARS-CoV-2 antigen. NPs can serve as excellent carriers for specific recognition molecules such as antibodies or probes as well as reporter molecules. Due to their high surface/volume ratio, they present more binding sites for capture elements and for reporting tags, leading to amplification of the analytical signal in a single recognition reaction.^33^ In the present study, AuNPs were synthesized via a physical route, using magnetron sputtering deposition on solid PEG substrate, and functionalized with anti-SARS-CoV-2 antibodies. The affinity and specificity of the bioconjugate were evaluated through comparative CLIA and ELISA assays.

We demonstrate the successful synthesis of AuNPs on solid substrates via magnetron sputtering, their stabilization, and their ability to conjugate with biomolecules. To the best of our knowledge, this is the first study to employ AuNPs synthesized on a solid substrate by magnetron sputtering in chemiluminescent diagnostic assays. Furthermore, we performed structural characterization using transmission electron microscopy (TEM), zeta potential (ZP), and UV–vis absorption spectroscopy, confirming the efficiency of the AuNP–antibody bioconjugate in immunoassays.

## Results and Discussion

### Synthesis and Characterization of AuNPs

AuNPs were synthesized through a magnetron sputtering process, resulting in AuNPs deposited on a dry polyethylene glycol substrate (Figure 1). This is a method that combines a straightforward AuNP preparation technique, offering low cost and storage stability, as the AuNPs remained stable for 12 months. The deposition time of 10 s proved effective for the process and application of this study, being significantly shorter than what is reported in the literature, which can take up to 50 min in liquid polyethylene glycol.^3^ Additionally, this method has the advantages of easy transport, stability, and no need for a controlled temperature storage. The PEG matrix in which the AuNPs are deposited is a well-known molecule because of its positive stabilizing effect on AuNPs in solution, providing an additional benefit.

**Figure 1:**
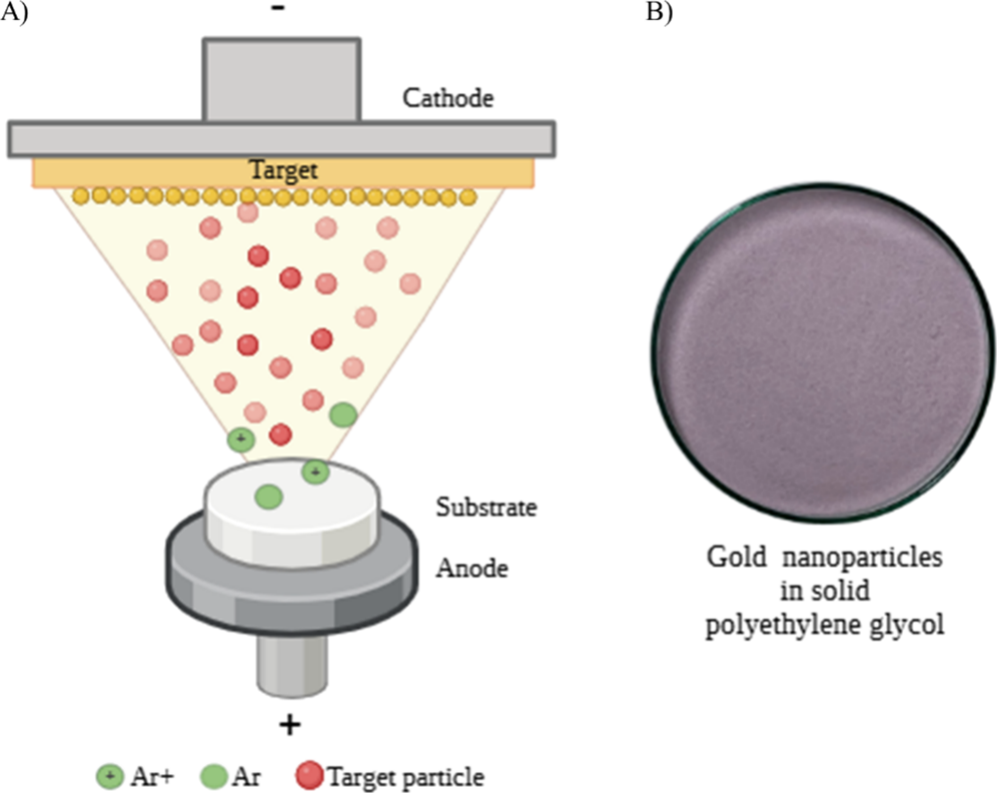
(A) Synthesis process of AuNPs using the top-down technique based on the magnetron sputtering method with deposition on a solid substrate. (B) AuNPs synthesized by magnetron sputtering on solid PEG.

The AuNPs were characterized using UV–vis spectrophotometry, X-ray diffraction (XRD), ZP, dynamic light scattering (DLS), and TEM with energy-dispersive spectroscopy (EDS). As shown in the UV–vis spectra in Figure 2, the plasmonic resonance band of Au can be observed with the corresponding Au element mapping (Figure 2F). The UV–vis characterization of AuNPs exhibited a λ max value of ∼535 nm (Figure 2A), corresponding to NPs around 50 nm in diameter, although the width of the band indicates size and shape dispersion, which was confirmed by TEM (Figure 2C–H).^35^ Additionally, the stability of the synthesis technique was confirmed after 12 months of dilution, where the colloidal solution of AuNPs obtained through magnetron sputtering deposition showed a λ_max_ of 537 nm. XRD spectrum for AuNPs reveals distinct diffraction peaks that confirm the crystalline structure of Au (Figure 2B). DLS analysis indicated that 55% of the AuNP sample had a size of 120 nm, and the ZP showed an average value of −7.34 mV for the AuNPs, which increased to −11.77 mV after functionalization. Typically, noble metal NPs, especially AuNPs, are stabilized in a citrate solution; however, stabilization with polyethylene glycol (PEG) is widely used to prevent AuNP aggregation.^36^ Since the NPs were deposited on PEG by magnetron sputtering, the remaining PEG is likely present even after removing excess PEG through centrifugation. Larger sizes observed in DLS and the lower ZP values might be due to the partial removal of the stabilizer from the sample.

**Figure 2:**
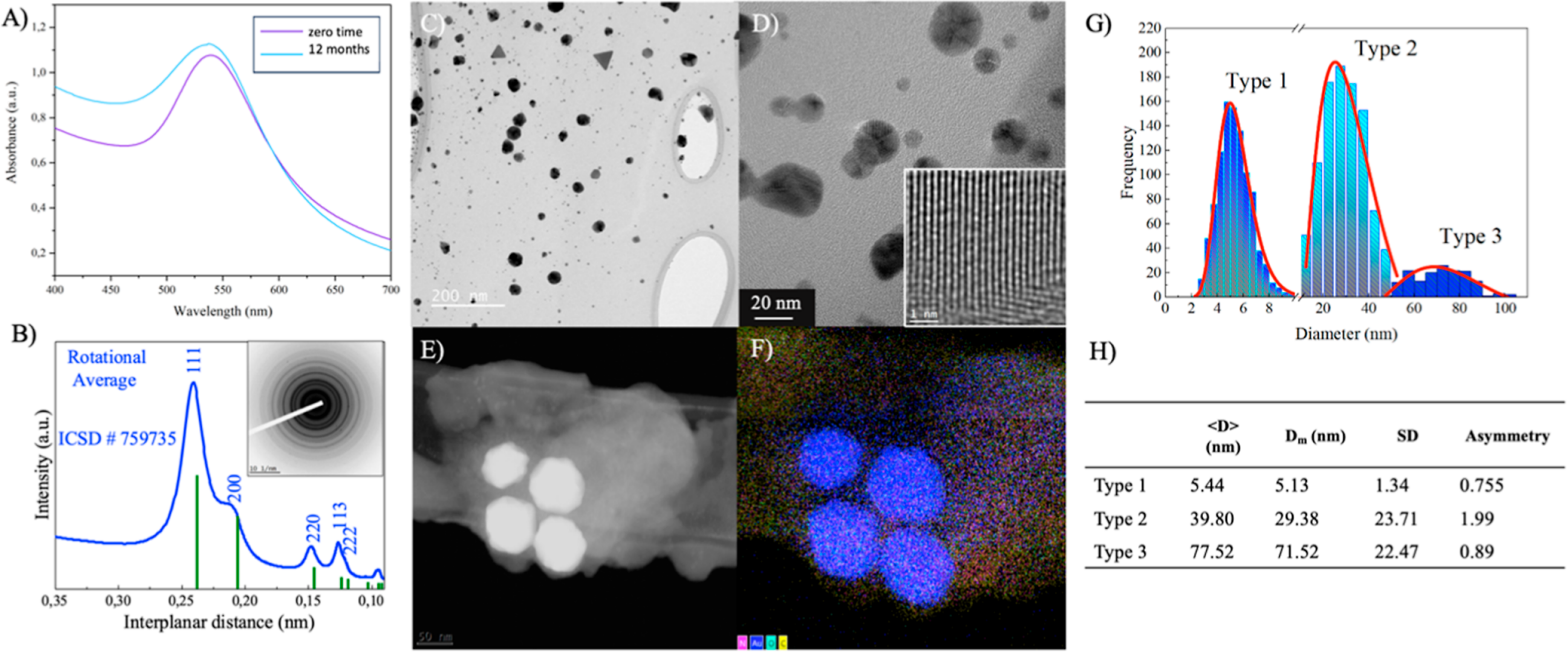
UV–vis characterization (A) of AuNPs synthesized by magnetron sputtering in PEG after dilution in ultrapure water at zero time and following 12 months. (B) XRD spectrum of AuNP. TEM analysis of AuNPs at low (C) and high (D) magnification. EDS spectroscopy mapping images of AuNPs (E) and the corresponding Au element mapping (F). Particle size distribution of AuNPs (G/H) with three distinct size types.

The morphology of the AuNPs was investigated using TEM (Figure 2C) and NPs exhibited a predominantly spherical morphology with an average diameter of ∼40 nm, which slightly increased to ∼45 nm after functionalization with antibodies. The EDS spectra recorded in the examined area show signals indicating the presence of Au atoms with the corresponding Au element mapping (Figure 2F), with traces of C, Si, and O atoms due to the presence of PEG (Figure 2F). The results presented here, using a solid substrate of PEG provided stable, easily transportable, low-cost, and viability of AuNP storage for long periods, at least 12 months were tested.

### Detection of SARS-CoV-2 N Protein

To develop a functional detection surface capable of capturing the target analyte, the surface chemistry of AuNPs was modified with SH-PEG3500-COOH, as PEG ligands are suitable and well-established for bioconjugation with a wide range of biomolecules.^37^ This modification enabled the coupling of an antibody with the enzyme peroxidase on the AuNPs, which aimed at capturing the SARS-CoV-2 N protein. Nucleocapsid proteins play a crucial role in viral replication and exhibit conserved regions within the CoV genus, being responsible for genome packaging and serving as a relevant diagnostic marker.^38^ Furthermore, the antibody against the N protein is shown to be more sensitive than the antibody against the spike protein.^39^

Due to the structural complexity of most antigens, multiple epitopes can be recognized by a broad repertoire of lymphocytes. Antibodies derived from a single B-cell clone are termed monoclonal and exhibit uniform affinity toward a specific epitope, whereas polyclonal antibodies consist of a heterogeneous mixture with varying affinities targeting multiple epitopes. To combine the advantages of both, we employed a pool of monoclonal and polyclonal antibodies for conjugation onto AuNPs, aiming to improve assay sensitivity and robustness (Figure 3A).

**Figure 3:**
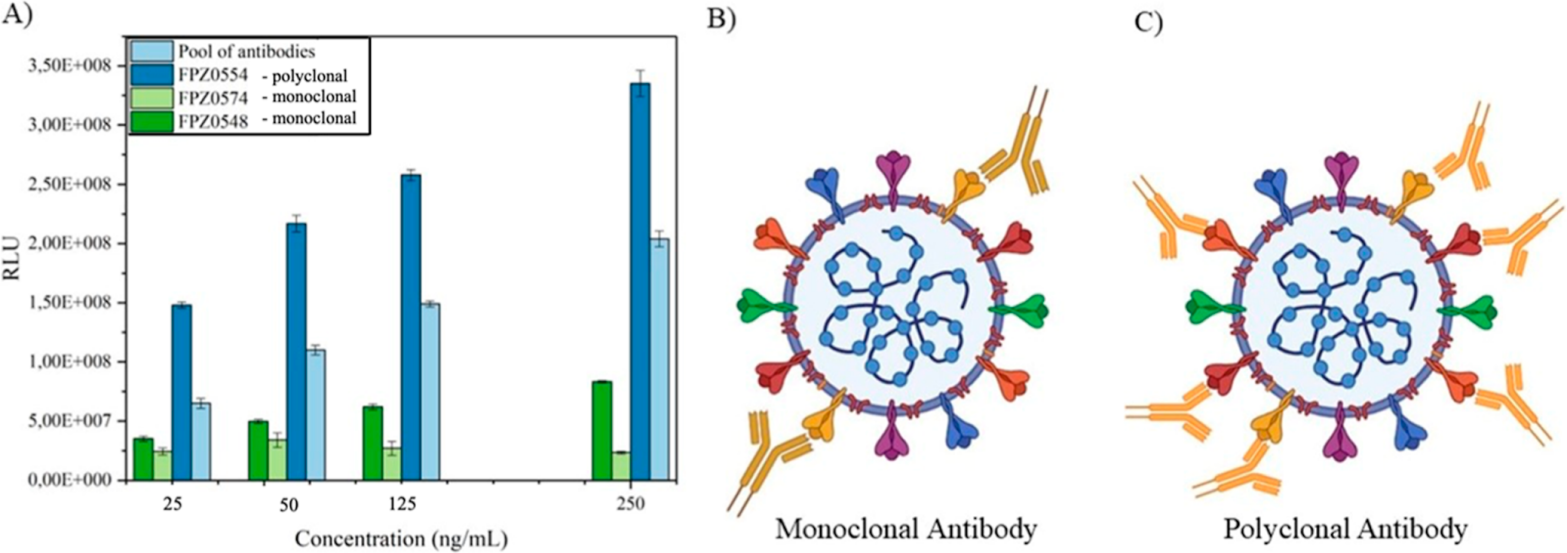
(A) Comparison of antibody sensitivity in CLIA assay: a pool of antibodies (FPZ0554—polyclonal, FPZ0574—monoclonal, and FPZ0548—monoclonal) are represented in light blue bars, showing an enhanced response of signal compared to FPZ0574, FPZ0548 antibodies individually, and lower affinity than FPZ0554 antibody; (B) representation of monoclonal antibodies binding to specific epitopes; and (C) representation of polyclonal antibodies binding to multiple epitopes.

To assess the affinity and specificity of the functionalized AuNPs, comparative experiments were conducted. Figure 4A illustrates the enhanced sensitivity of the ELISA immunoassay at higher antigen concentrations (>150 ng/mL) in the sample; however, this signal amplification does not occur at lower concentrations. In contrast, in the CLIA (Figure 4B), low antigen concentrations (<150 ng/mL) exhibit an increased signal conjugated to AuNP, indicating an enhancement in sensitivity for samples that could have been inaccurately detected as negative.

**Figure 4:**
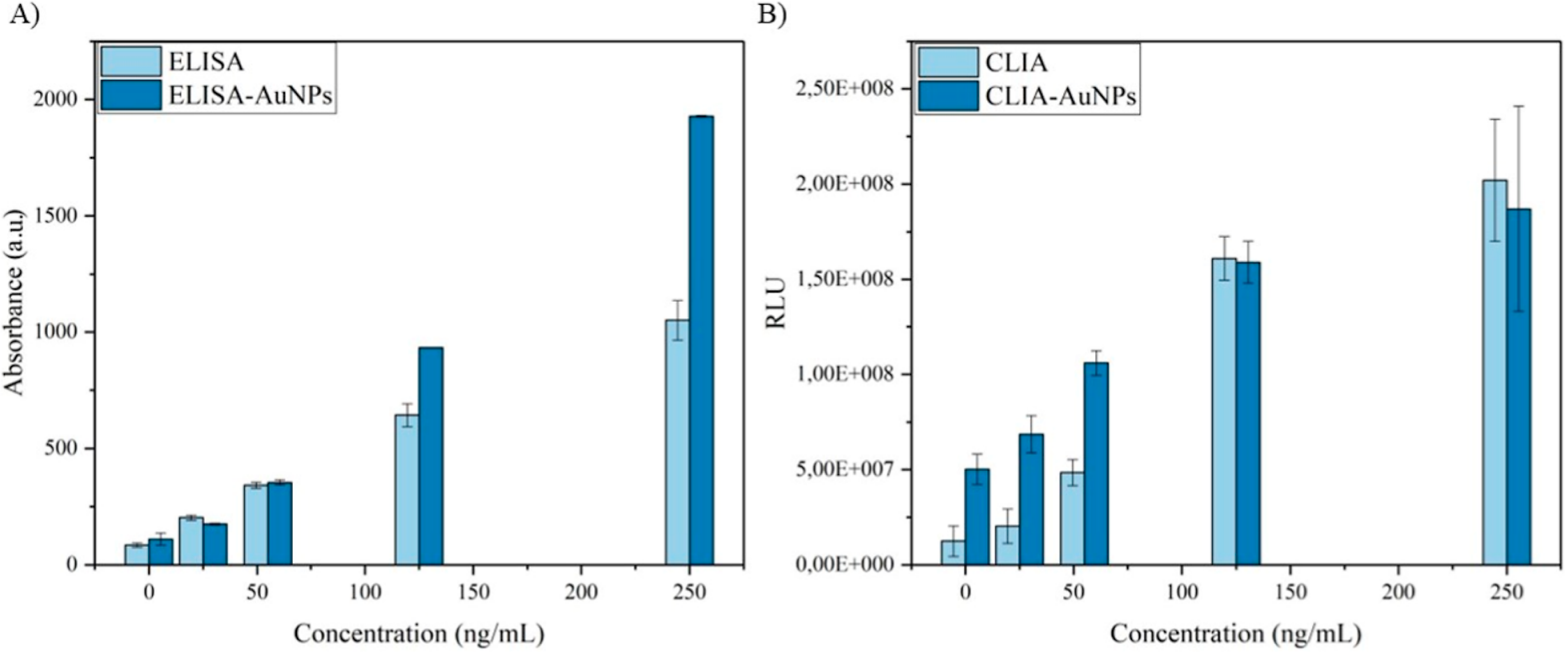
Comparison of (A) ELISA and (B) CLIA with and without AuNPs. An enhanced sensitivity of the ELISA immunoassay is seen at higher antigen concentrations (>150 ng/mL), in contrast to the CLIA system, where presence of AuNP is amplified at low concentrations (<150 ng/mL).

### Chemiluminescent Assay Using AuNPs and Clinical Samples

Human biofluids possess complex compositions compared to that of a single solute solution. Therefore, to assess the reliability of AuNPs in clinical applications, we conducted a CLIA based on AuNPs using 180 nasopharyngeal samples collected during the COVID-19 pandemic. The assay was analyzed using the Receiver Operating Characteristic (ROC) curve, which indicated the optimal cutoff value of 1.19 ng/mL for AuNP-CLIA. The results of the ROC curve analysis for COVID-19 are presented in Figure 5. The ROC curve analysis for AuNP-CLIA (Figure 5A) revealed an AUROC of 0.909 (95% CI 0.864–0.955), demonstrating a sensitivity of 91% and a specificity of 85%, as summarized in the 2 × 2 table (Figure 5 B).

**Figure 5:**
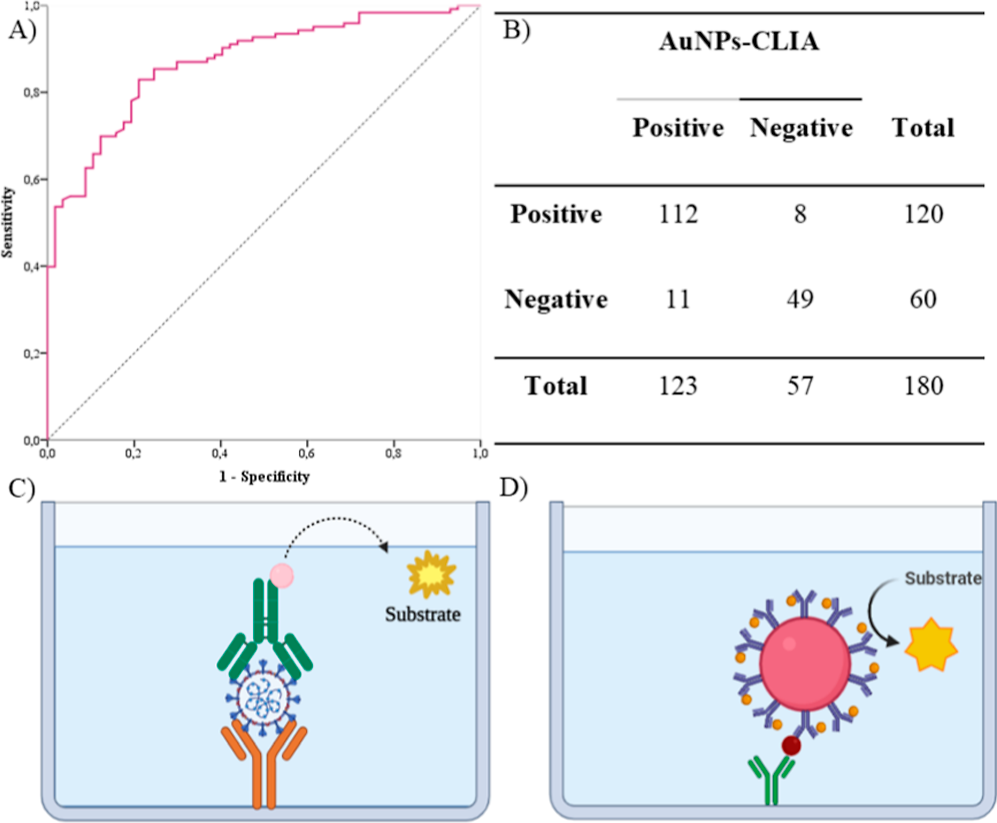
(A) ROC curve of the AuNP-CLIA for 180 samples of patients with COVID-19 compared to healthy individuals; (B) results obtained from nasopharyngeal samples of patients using the AuNP-CLIA technique for the detection of SARS-CoV-2 antigens; (C) traditional sandwich immunoassay; and (D) sandwich immunoassay based on AuNPs as carriers of antibodies and enzymes.

Traditional ELISA, while being a simple, sensitive, and reliable tool, is not free from limitations. Therefore, to enhance the immunoassay, several strategies have been developed that can be broadly categorized into non-nanotechnology-based approaches (such as chemiluminescent assays) and nanotechnology-based approaches (involving the application of AuNPs).^40,41^

Among the strategies to improve traditional immunoassays, one of the primary functions of AuNPs is to serve as carriers for antibodies and enzymes to amplify the signal. In traditional immunoassays, one limiting assay sensitivity is the low enzyme/antibody ratio, typically 1:1 (Figure 5C). To address this, a strategy to enhance the signal is to increase the number of enzyme molecules in the final antigen–antibody-enzyme complex.^40^ Consequently, the AuNP-CLIA approach facilitates signal optimization by utilizing AuNPs functionalized with the anti-SARS-CoV-2 antibody and HRP enzyme (Figure 5D), thereby increasing the aforementioned ratio. The ability to immobilize multiple enzymes and antibodies on the surface of a single NP can significantly enhance the sensitivity of immunoassays.

Diagnostic research for COVID-19 through immunoassays generally detect anti-SARS-CoV-2 antibodies, especially IgM antibodies, after 3 days of infection, reaching peak levels within 2 to 3 weeks. Detection of IgG antibodies may also be pursued in the absence of IgM antibodies for up to 3 months postinfection.^42^ Consequently, it has been reported that immunological tests do not assist in diagnosing and tracking early infection;^26^ however, the identification of IgM antibodies indicates a current infection, while IgG antibodies suggest a prior infection.^43,44^ In our study, the AuNP-CLIA demonstrated accuracy in identifying symptomatic patients early in infection by detecting the SARS-CoV-2 N viral protein.

ELISA-type immunoassays tend to show improved sensitivity as time increases following the onset of infection, considering the average viral incubation period, which is approximately 4 to 6 days.^45^ Recently, our research group has published a paper comparing in-house immunoassay for ELISA and CLIA to diagnose COVID-19 in nasopharyngeal samples.^46^ The results showed that CLIA was able to detect active disease in samples containing N protein concentrations greater than 16 ng/mL, with a sensitivity of 90% and specificity of 94% in contrast to the ELISA system, with sensitivity at 54% and specificity at 87%. Although ELISA offers very similar advantages, the best results for accuracy were observed for the in-house CLIA system. It has already been reported that CLIA exhibits superior sensitivity at lower antigen concentrations.^47^ Regularly, CLIA shows better results in terms of sensitivity and specificity.^45^ Regarding AuNP-based immunoassays, their capacity to immobilize multiple enzymes and antibodies on the surface of a single NP allows for a significant increase in immunoassay sensitivity and an expanded detection range without complex synthesis procedures.^40^

## Conclusion

The results obtained in this study highlighted the importance of investigating and developing environmentally safe AuNP synthesis for biotechnological applications. In this study, we developed a new route of synthesis using a physical method, where no chemical residues are left after processing. We also characterized these AuNPs and showed that these nanomaterials are stable and prone to functionalization using immunological approaches once AuNPs can be utilized as carriers for antibodies and enzymes. The ability to immobilize multiple enzymes and antibodies on the surface of a single NP significantly enhances the sensitivity of the immunoassays. Additionally, the AuNPs synthesized by magnetron sputtering exhibited promising characteristics, enabling their use for various approaches, including the diagnosis of viral diseases. Regarding the AuNP-CLIA, it was possible to achieve optimal sensitivity and specificity for SARS-CoV-2 in samples collected throughout the pandemic period. Thus, the immunoassay proved to be an excellent tool for diagnosing the SARS-CoV-2 viral protein and can be utilized for patient screening systems in a variety of infectious diseases.

## Experimental Section

### Clinical Samples

This study was approved by the Research Ethics Committee (36111720.3.0000.5341) at the Universidade de Caxias do Sul (UCS). A total of 273 individuals attended the clinical center (CECLIN) of the Universidade de Caxias do Sul/RS, seeking nasopharyngeal swab testing for RT-PCR at the specific COVID-19 service between 2020 and 2022. These patients signed a consent form, allowing their samples to be tested using immunological assays and compared with standard samples collected via nasopharyngeal swabs.

### Sample Collection

Each participant obtained the sample through a nasopharyngeal swab collection performed by a trained professional. A swab was gently inserted into the patient’s right nostril until resistance was felt at the pharynx. The swab was held in place for a few seconds to allow for the absorption of secretions. It was then rotated for approximately 20 s. Afterward, the swab was slowly removed, and the process was repeated with the same swab in the patient’s left nostril. Following the collection from both nostrils (right and left), the swab was immediately placed in a tube containing 3 mL of PBS and stored at −80 °C until analysis. The inclusion criteria for samples were as follows: epidemiological history, clinical symptoms (fever, cough, dyspnea, alteration of taste and smell, and contact with an infected individual), and RT-qPCR testing. A total of 97 samples were excluded due to the following factors: CT value > 30, indeterminate RT-qPCR result, and insufficient sample quantity for conducting all diagnostic tests, leaving 180 samples that met all the requirements for the study.

### Synthesis of AuNPs via Magnetron Sputtering

The PEG compound with a molecular weight of 8000 g/mol (Sigma-Aldrich, US) was utilized as a substrate for the magnetron sputtering of high-purity Au. The process was carried out in a vacuum chamber with a base pressure of 3 × 10^–6^ mbar and a working pressure of 4 × 10^–3^ mbar. Following deposition, the mixture was diluted in deionized water in a ratio of 1:10 (PEG-Au/water) and stored at room temperature.

### Characterization of AuNPs by UV–vis, TEM, and EDS

The characterization of the colloidal solutions of AuNPs was conducted after synthesis using molecular absorption spectroscopy in the visible region, DLS, ZP analysis, and TEM.

For UV–vis analysis, readings were performed using a spectrophotometer (UV-2600i Shimadzu, Japan) in the wavelength range of 350 to 750 nm, with measurements taken at 1 nm intervals. The analyzer was programmed to perform 5 cycles with 15 repetitions for each sample for the characterization of AuNPs (DLS Zetasizer Nano Series, Malvern). For ZP analyses, the same sample preparation and equipment as previously mentioned were employed, utilizing the Zetasizer Nano (Malvern, UK).

The size distribution of the AuNPs was measured using ImageJ software based on images captured during the TEM analysis (JEOL 2100F) with CCD, EDS, EELS, and Precession Diffraction.

### Functionalization of AuNPs with Anti-SARS-CoV-2 Antibodies

The AuNPs were first centrifuged at 7000 g. Subsequently, HS-PEG-COOH diluted in deionized water was added and incubated for 1 h. The solution was then centrifuged again and activated with EDC/NHS (in a 1:10 ratio) for 20 min, followed by washing with deionized water. Afterward, a pool of polyclonal and monoclonal antibodies against the nucleocapsid of SARS-CoV-2 (Fapon Biotech, China) was incubated for 1 h. Finally, the bioconjugate AC-PEG-AuNP was washed with a blocking solution containing BSA, PEG-HS, and 50 mM Tris and stored at 4 °C.

### Standardization of the Diagnostic Test–Detection of the Target Protein

The CLIA was performed by incubating the monoclonal nucleocapsid antibody FPZ0638 (Fapon Biotech, China) diluted in carbonate-bicarbonate buffer (50 mM, pH 9.6) at 37 °C in Immuno Standard Modules White plates with MaxiSorp surface treatment (437591, Thermo Scientific, US). Following this, a blocking step with 1% BSA was conducted; from this stage onward, the test was carried out at a temperature of 21 °C. Subsequently, biological samples from nasal swabs and the Ac-AuNP bioconjugate solution were added to the wells and incubated for 1 h. The revelation step was performed using the SuperSignal ELISA femto substrate kit diluted in deionized water at a ratio of 1:1 (Thermo Scientific, US), and the intensity of the emitted light was measured after 10 min of incubation at 470 nm using a luminometer (SpectraL–Molecular Devices, US). The test was performed in duplicate. Assays were accomplished with a new calibration curve using recombinant protein FPZ0516 (Fapon Biotech, China).

Calculations for sensitivity (eq 1) and specificity (eq 2) were performed based on a 2 × 2 table, in which the results were categorized as true positives, false positives, true negatives, and false negatives, using the equations below:

1 $$Sensitivity=\frac{TP}{TP+FN}$$

2 $$Specificity=\frac{TN}{FP+TN}$$

A total of 180 clinical nasopharyngeal swab samples were tested for CLIA with the addition of AuNP.

### Statistical Analysis

The ROC curve and the 95% Clopper-Pearson confidence intervals were calculated for sensitivity and specificity using the IBM SPSS statistical software package, version 20.
